# In vivo Labeling of Bone Microdamage in an Animal Model of Type 1 Diabetes Mellitus

**DOI:** 10.1038/s41598-019-53487-6

**Published:** 2019-11-18

**Authors:** Sahar Mohsin, Suneesh Kaimala, Eman Khamis Yousef AlTamimi, Saeed Tariq, Ernest Adeghate

**Affiliations:** Department of Anatomy, College of Medicine and Health Sciences, United Arab Emirates University, Al Ain, PO Box. 17666 UAE

**Keywords:** Type 1 diabetes, Bone

## Abstract

Type 1 diabetes mellitus (DM1) is linked to a decrease in bone strength. Bone strength entails both bone mineral density and bone quality. Limited data are available regarding diabetes-induced microdamage, which can severely influence bone quality. This study has investigated bone microdamage as a measure of bone quality in an animal model of DM1. Microdamage in the neck of the femur was labelled *in vivo* using multiple fluorochromes at 4, 12 and 24 weeks after the onset of DM1. Microcracks were quantified and their morphology analyzed using microscopy techniques. The mean length of microcracks at 24 weeks, and crack numerical and surface densities were significantly higher (p < 0.05) 4 weeks after the onset of DM1 when compared with control. Diffuse damage density was highest at 12 weeks after the onset of DM1. The arrangement of the collagen fibrils became progressively more irregular from 4 to 24 weeks of DM. This is the first study to analyze microdamage *in vivo* at different time points of DM1. DM1is associated with microcracks from the early stage, however bone microstructure shows toughening mechanisms that arrest their growth but disease progression further deteriorates bone quality resulting in longer microcracks which may increase fracture risk.

## Introduction

Diabetes mellitus (DM) is regarded as one of today’s most pressing health care challenges adversely affecting multiple organs in the body with increasing morbidity and mortality rates globally. Diabetes and its complications bring about a substantial financial burden to national health care systems. The prevalence of DM has been steadily increasing over the past few decades. Globally, an estimated 422 million adults were living with diabetes in 2014, compared to 108 million in 1980^1^.

Type 1 DM (DM1) occurs mostly in childhood due to deficient insulin production by the pancreas resulting in hyperglycaemia. DM1 may also occur in adults and predisposes individuals to a greater risk of fractures. The higher fracture incidence in DM1 patients with respect to the general population is not only due to an increased tendency to falls as a result of its complications such as peripheral neuropathy, poor vision and stroke and but also due to early osteoporotic changes resulting in increased bone loss and/or altered bone matrix and strength^2,3^.

Bone strength is dependent on both “bone quantity” and “bone quality”^4^. Bone quantity is a measure of bone mineral density (BMD) and is routinely measured by dual-energy X-ray absorptiometry (DXA) scan in the clinics. Osteoporosis is diagnosed when the BMD is less than or equal to 2.5 standard deviations or more below the average value for young adults (a T-score of <−2.5 SD)^5^.

Osteoporosis is a disease characterized by abnormalities not only in the amount (bone quantity) but also in architectural arrangement of bone tissue (bone quality) that leads to impaired skeletal strength and undue susceptibility to fractures^6^. It is a silent disease as there are no warning signs until one experiences a fracture after a minor fall, which otherwise in a healthy adult would not have led to a fracture^7^. The bone quality factor for osteoporosis entails bone microarchitecture, bone turnover, mineralisation, the extent of microdamage and the composition of bone matrix and mineral^4,8,9^.

The risk of fractures is significantly greater in type 1 DM when compared to the general population^3^. Most of the studies in the past have linked type I DM with osteoporotic changes by defining only bone quantity but the measurement of bone quantity alone does not always reliably predict fracture risk^3^. This has stimulated us to investigate aspects other than bone quantity that contribute to bone fragility.

Microdamage, in the form of microcracks or diffuse microdamage, is an important determinant of bone quality in osteoporosis. The origin of damage at the ultrastructural level is not known. Bone microdamage is investigated in multiple forms across the scales of hierarchy in bone^10^. It is hypothesized that the damage at the ultrastructural level is caused by the cracking of bone mineral crystallites, debonding at the mineral organic interface or shearing between and within collagen fibrils^11–13^.

Microcracks act as a stimulus for bone remodeling and are repaired by “targeted” remodeling to the sites of damage^14–16^. The generation of microcracks within the bone matrix is regarded as a protective mechanism to dissipate energy and avert fracture. However, if damage accumulates beyond the bone’s capacity to repair due to an imbalance of bone remodeling process it contributes to the reduced toughness of both cortical and trabecular bone and ultimately leads to fragility fractures as in ageing or as a result of various disease processes^16^.

Some studies^17,18^ did look at the alterations in the microstructure of bone in type 1 DM but there is a lack of data investigating the microdamage which is yet another important factor in determining bone quality. Microdamage accumulates and increases with ageing and we hypothesize that DM1 being a chronic disease may reduce bone quality over a period of time and so, in this study we analyzed microdamage at different intervals since the onset of diabetes.

Microcracks were detected in human bones *in vitro* by Frost^19^ using basic fuchsin dye. Most of the studies labeled microdamage *in vitro* using the en bloc fuchsin method^19^ but fuchsin dye is not site-specific so it labels microcracks but also other voids and spaces within bone tissue^20^. Only a few studies have used an *in vivo* labeling technique to assess bone microdamage^21^ and this is the first study to our knowledge to label microdamage *in vivo* at the different duration of DM using fluorescent chelating agents.

Fluorochromes are calcium-binding substances that label all the active sites of mineralisation including microcracks. Three different florescent chelating agents are used in this study; alizarin to label the pre-existing damage and calcein and xylenol orange to label microcracks *in vivo* at the different duration of DM. We have analyzed the microcracks *in v*ivo at 4, 12 and 24 weeks of the duration of diabetes. Previously Mohsin *et al*.^22^ have used fluorescent chelating agents to sequentially label microcracks *in vitro* and have investigated the effect of crack length on its propagation through the microstructure of bovine compact bone.

The aim of the current study was to investigate microdamage in streptozotocin-induced type 1 diabetic rats. Microdamage was detected, quantified and its behaviour in trabecular bone was analysed at different time points.

## Results

**Figure 1:**
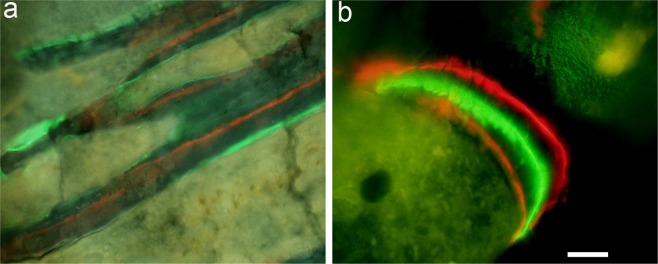
(**a**) actively growing surfaces are labeled with calcein (green) and xylenol orange (orange); (**b**) all three labels alizarin (red), calcein (green) and xylenol orange (orange) are visible when viewed under the fluorescence microscope with combination filter cube ET-DAPI/FITC/TRITC 69000. Scale bar represents 200 µm for (**a**) and 100 µm for (**b**).

**Figure 2:**
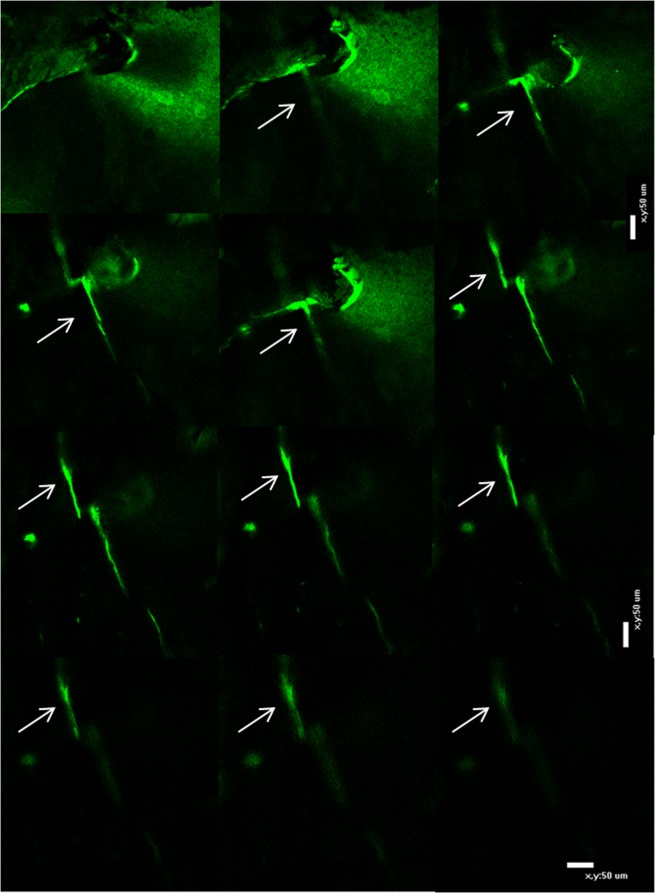
(**a**) actively growing surfaces are labeled with calcein (green) and xylenol orange (orange) white arrow. Linear wavy microcrack stained with calcein is also shown (black arrow) (**b**) linear microcrack stained with calcein shows discrete bridging pattern (**c**) thin wavy microcrack (white arrow) stained with xylenol orange. (**d**) tortuous microcrack stained with calcein showing a frontal zone process at its tip (white arrow) (**e**) linear microcrack and diffuse damage stained with calcein shown by white and red arrows, respectively (**f**) Microcracks deflect or split (**g**) as it comes across a microstructural feature (**h**) calcein stained linear microcrack showing a discrete bridging pattern (**i**) obliquely placed microcrack showing a discrete bridging behaviour stained with xylenol orange (**j**) propagating microcracks stained with calcein (white arrow) and xylenol orange (red arrow). Images are taken by a combination filter cube ET-DAPI/FITC/TRITC 69000 for a, e, h, i, j. FITC for images b, d, f, g and TRITC for image c. Scale bar represents 200 µm for a,b,i,j; 100 µm for e,f,g,h and 50 µm for c and d.

The fluorescent agents labelled all the actively growing surfaces (Figs 1a,b and 2a) from where mineral apposition rate was calculated at different time points and data obtained showed decreased values for all diabetic samples compared to the non-diabetic ones (Table 1). The microdamage was also labelled as red, green and orange for alizarin, calcein, and xylenol orange, respectively when viewed under the fluorescence microscope with a combination filter cube ET-DAPI/FITC/TRITC 69000 (Fig. 2). We found microcracks labelled with alizarin but we did not quantify those microcracks as they represent pre-existing damage before the induction of DM.

**Table 1:** Microcrack data analysis showing the mean ± standard deviation for microcrack length (Cr.Le), crack numerical density (Cr.N.Dn), crack surface density (Cr.S.Dn) and diffuse damage density (Dx.Ar) in the neck of the femur from Wistar rats in the controls and type 1 diabetes at four (A-DM), twelve (B-DM) and twenty-four (C-DM) weeks of duration of diabetes.

Mineral Apposition Rate (MAR) µm/day					
Control			Diabetic		
A 4 week	B 12 week	C 24 week	Gp A-DM 4 week	Gp B-DM 12 week	GP C-DM 24 week
29.6 ± 4	25.2 ± 2.8	16.8 ± 3.9	16.7 ± 3.6**	12.7 ± 2.5 **	11.89 ± 4.8

**(p < 0.01) GP A- GpA-DM; GpB- Gp B-DM.

**Figure 3:**
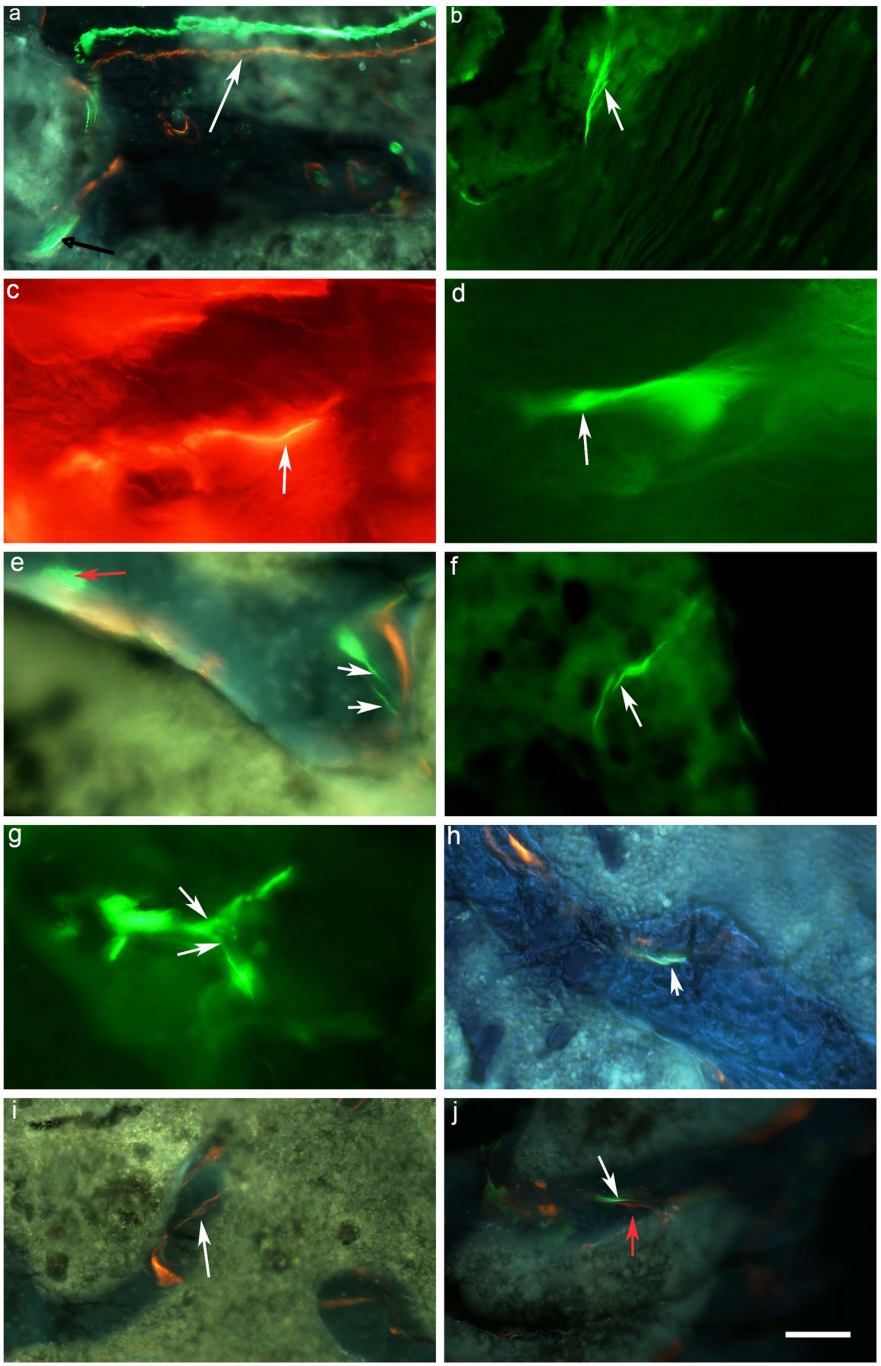
Series of the image acquired using a confocal microscopy showing microcrack traced throughout the depth of a section (arrows). Bar: 50 µm.

Confocal microscopy was used to confirm that microcracks were labelled throughout the entire depth of the section (Fig. 3) and quantitative data was obtained using fluorescence microscopy.

**Figure 4:**
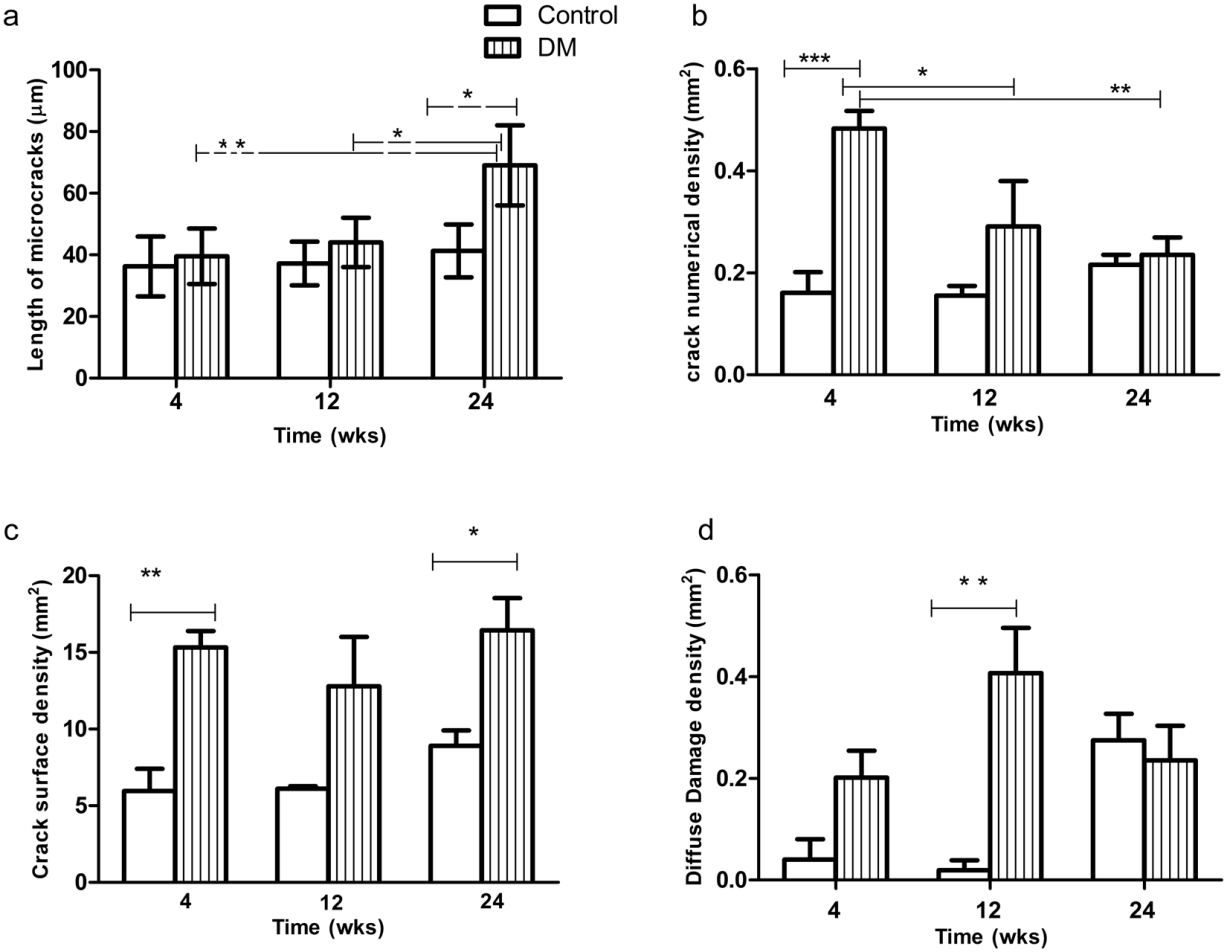
(**a**) Mean length of microcracks at 4, 12 and 24 weeks in control and animals with type 1 diabetes mellitus. Longer microcracks were found at the 24-week onset of diabetes when compared to the control group [p < 0.05 indicated by (*)] and with 4 and 12-week onset of diabetes p < 0.01 (**) and p < 0.05; respectively (**b**) Shows crack numerical density (Cr.N.Dn) in controls and rats with 4, 12 and 24 weeks of the onset of type 1 diabetes mellitus. Cr.N.Dn is significantly [p < 0.001 (***)] higher at 4 weeks when compared with control and with 12 weeks [p < 0.05 (*)] and 24 weeks (p < 0.01) duration of diabetes (**c**) Crack surface density is significantly [p < 0.01 (**)] higher at 4 weeks and 24 weeks [p < 0.05 (*)] of duration of diabetes when compared with their respective controls (**d**) Diffuse damage density is significantly [(p < 0.01)] higher at 12 weeks of duration of diabetes compared to control.

Areas of diffuse damage were observed as a collection of multiple smaller microcracks 1–10 µm long (Fig. 2e). The number of diffuse damage area (Dx.Ar) were significantly (p < 0.01) higher 12 weeks after the onset of DM when compared to control (Fig. 4d).

### Pattern of microcracks

We analyzed the interactions of linear microcracks with the microstructure of bone. Microcracks were found in the trabecular bone of the neck of the femur. We were able to identify various patterns of microcracks;

- Thin and wavy along the direction of collagen fibres (Fig. 2a,c,d).
- Discrete cracking or bridging (Fig. 2b,h,i).
- Deflected by the microstructural feature such as Haversian canal, lacunae or resorption cavities (Fig. 2f).
- Split as they come across a change in the direction of collagen fibres or hit some microstructural feature (Fig. 2g).
- Transverse or oblique within or between two trabaculae (Fig. 2i).

### Course and direction of microcracks

Some of the microcracks showed a tortuous course along the direction of collagen fibres and microcracking at the tip (Fig. 2d). We also observed a few linear microcracks perpendicular to the direction of lamellae and observed as transverse or oblique microcracks (Fig. 2i). However, the majority of microcracks were present longitudinally along the direction of collagen fibres in all the samples. We also found the microcracks stained by multiple dyes as shown in Fig. 2j; a microcrack labelled by calcein and xylenol orange dyes.

### Quantitative data

Mean crack length is affected by the duration of the DM. Microcracks were found to be much longer in the neck of the femur of Wistar rats after 24 weeks’ duration of diabetes compared to control p < 0.05 and were longer when compared to A-DM and B-DM groups p < 0.01 and p < 0.05, respectively (Fig. 4a). Table 2 entails the mean length of microcrack (Cr.Le) µm), crack numerical density (Cr.N.Dn), crack surface density (Cr.S.Dn), and diffuse damage density (Dx.Dn). Statistical analysis indicates that DM1 affects bone microstructure. Crack numerical density was significantly higher (p < 0.001) at 4 weeks of the onset of diabetes when compared with its respective control and with 12 (p < 0.05) and 24 (p < 0.01) weeks after the onset of DM (Fig. 4b).

**Table 2:** Mineral Appositional rates (mean ± standard deviation) is shown in the neck of the femur from Wistar rats in the controls and type 1 diabetes at four (A-DM), twelve (B-DM) and twenty-four (C-DM) weeks of the duration of diabetes.

	Control			Diabetic		
	A 4 week	B 12 week	C 24 week	Gp A-DM 4 week	Gp B-DM 12 week	GP C-DM 24 week
Mean length of microcracks (Cr.Le) µm	36.25 ± 9.7	37.22 ± 7.1	41.27 ± 8.6	39.52 ± 9.3	44 ± 8.78	69.81 ± 13.5
Mean crack numerical density (Cr.N.Dn) (mm^2^)	0.16 ± 0.07	0.15 ± 0.03	0.21 ± 0.03	0.48 ± 0.06	0.29 ± 0.15	0.23 ± 0.05
Mean crack surface density (Cr.S.Dn) (mm^2^)	5.95 ± 2.52	6.11 ± 0.28	8.91 ± 1.7	15.32 ± 1.8	12.79 ± 5.6	16.44 ± 3.6
Mean diffuse damage density (Dx.Dn) (mm^2^)	0.04 ± 0.06	0.04 ± 0.03	0.3 ± 0.08	0.20 ± 0.01	0.41 ± 0.15	0.23 ± 0.11

Cr.S.Dn was highest in GP A-DM (p < 0.01) and in Group C-DM (p < 0.05) when compared to all controls and Group B-DM (Fig. 4c).

### Collagen fibrils in bone

**Figure 5:**
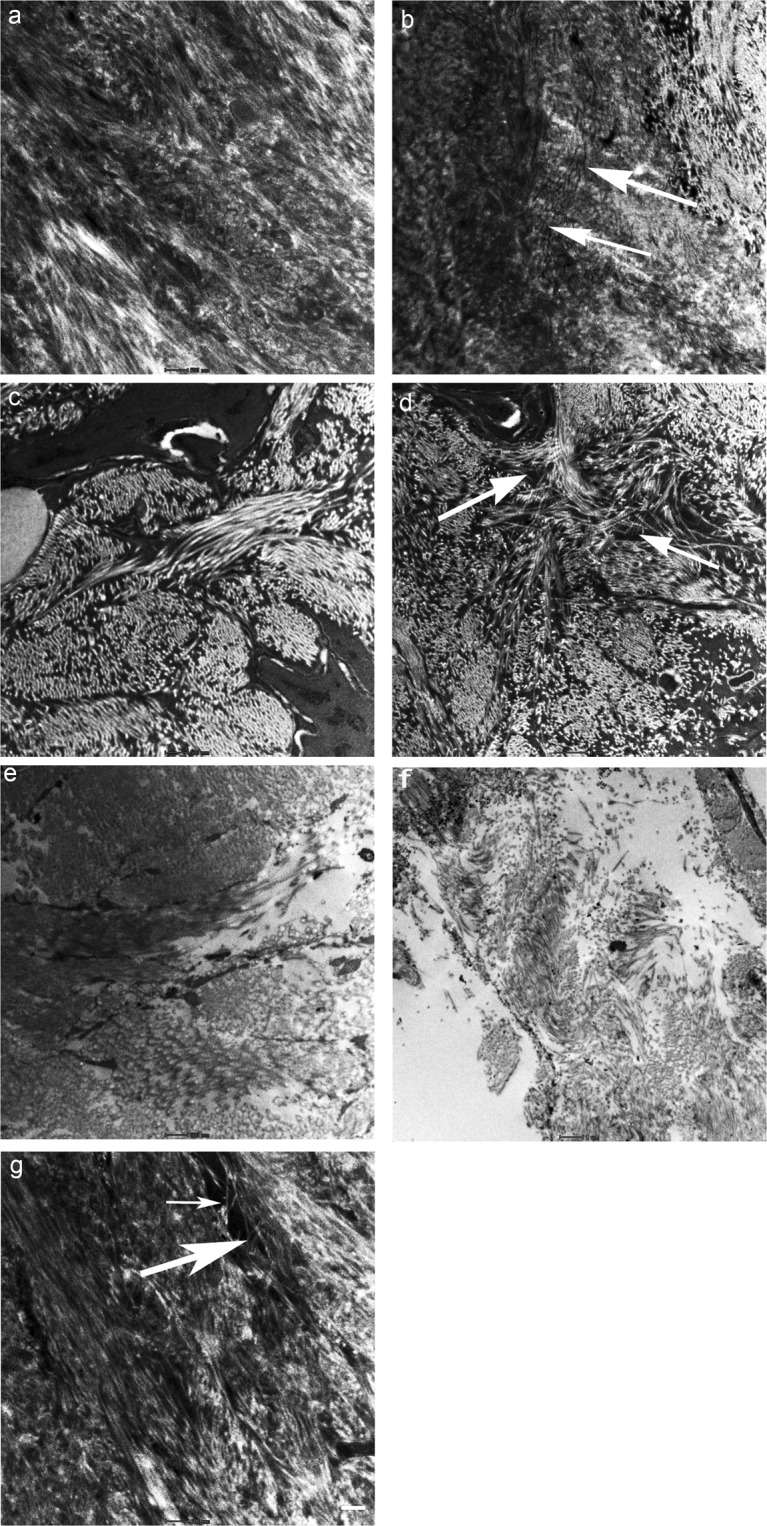
Transmission electron microscopy of the arrangements of collagen fibres in control and bone samples from 4, 12 and 24 weeks after the onset of diabetes (**a–f**). Bone specimen from control group at 4 weeks shows tight packing of collagen fibrils (**a**) and in experimental group (**b**) shows initiation of ultrastructural changes resulting in debonding of collagen fibrils and formation of linear microcracks (arrows) (**c**) Bundles of collagen fibrils are arranged in different directions in bone specimens from controls at 12 weeks (**d**) collagen fibrils forming a meshwork (arrows) enclosing various porosities in bone specimens at 12 weeks of duration of diabetes (**e**) collagen fibrils and bone matrix are more compact in control animals at 24 weeks (**f**) completely disorganized arrangement of collagen fibril number and size of afibrillar voids increases in bone specimens from 24 weeks of the duration of diabetes (**g**) showing microcrack bridging pattern (arrows). The white scale bar is 500 nm for (**a–c** and **e,g**) and 1 µm for (**d,f**).

The TEM study shows that the arrangement of the collagen fibrils becomes progressively irregular from 4 weeks to 24 weeks of diabetes (Fig. 5b,d,f). Collagen fibrils were tightly packed in bone specimens from control animals (Fig. 5a,c,e). Numerous microcracks appeared as debonding of collagen fibrils starting at an early phase of diabetes (Fig. 5b). Figure 5c shows an arrangement of collagen fibrils in different directions. The interlacing of collagen fibrils has created a meshwork with loose pockets interspersed with normal fibrils at various areas in bone specimens (Fig. 5d). At 24 weeks, changes in the arrangement of collagen fibrils were seen in bone samples from both control and diabetic animals. The disarray of collagen fibrils became more pronounced in 24-week post diabetic samples. Collagen fibrils were arranged in a disorganized/random manner with much bigger gaps between the collagen fibrils at 24 weeks of the duration of diabetes. The bridging pattern of microcracks observed in fluorescence microscopy (Fig. 2b,h,i) was also seen with TEM (Fig. 5g).

## Discussion

Bone fragility is a well-known complication of type I DM aggravating osteopenia and osteoporosis^3^ but patients with type 1 DM have a fracture risk much higher than expected, which cannot be explained by their reduced bone mineral density alone^3,23^. The current study, therefore, has investigated the bone quality factor for bone fragility in type I diabetes by focusing on the assessment of microdamage in the femoral neck of adult male Wistar rats by labeling it with fluorescent chelating agents *in vivo* at the different duration of diabetes. Dyes were injected in decreasing order of affinity for calcium hence alizarin was applied first to label the pre-existing damage before the induction of experimental diabetes^24^. The bone microstructure was further investigated using transmission electron microscopy.

Two types of damage were found in bone samples; linear microcracks and diffuse damage. Linear microcracks were identified as per standard criteria^25^ and can be seen as cracks with sharp borders larger than canaliculi but smaller than vascular channels. They were stained through the thickness of the section and on changing the depth of focus, crack edges appeared more sharply stained than the rest of the crack. Diffuse damage is a collection of small sub-micron size microcracks size (<1 μm)^26^ and was seen as a pooling of dye-labeled with calcein or xylenol orange or by both dyes under a fluorescence microscope. Both types of damage contribute independently to bone matrix quality^26^. Microcracks found in this study were also further confirmed by the use of LSCM that they are stained throughout the depth of the section. LSCM is a combination of fluorescence microscopy, laser light illumination, and computer image processing. The advantage of this technique over conventional microscopy methods is that it shows a much improved spatial resolution because the laser can be focused with high precision onto the actual microcrack being examined and this allows the elimination of the out-of-focus image from the image focal plane^27^. However, we were unable to obtain a sufficient number of thicker ground sections for diabetic bone specimens as bone tend to break during the grinding process, therefore, we used only thinner sections for obtaining quantitative data.

The majority of the linear microcracks found in this study were thin and wavy. This observation corroborates those reported by Zioupos and Currey^28^. They were present in the older interstitial bone between the trabeculae and are oriented longitudinally. We also found microcracks that lie perpendicularly or at an angle to the direction of collagen fibres within trabaculae. A number of microcracks forming a bridging pattern were also observed. Bridging elements decrease the stress intensity near the crack tip and thus toughens the brittle material^29^. However, a typical cross-hatched pattern microcrack reported in earlier studies^30^ was not found in this study. We found microcracks deflected or split as they come across a microstructural feature such as a blood vessel, lacunocanalicular porosity, and a resorption cavity or as the lamellae within trabeculae changes direction. Hence microstructural features such as number, structure and direction of the trabaculae, lacunocanalicular network, presence of blood vessels and arrangement of the collagen fibres within lamellae were the main factors determining the behaviour of microcracks in the trabecular bone^31,32^ rather than secondary osteons bounded by cement lines as seen in compact bone^22^.

Tortuous microcracks forming a frontal process zone i.e micro-cracking around the main crack tip followed by the formation of a wake zone which contains microcracks that are left behind as the crack propagates into the matrix as a linear microcrack were observed in this study. This mechanism causes a reduction in the stress intensity around the crack tip and is regarded as one of the toughening mechanisms which occur due to the anisotropic structure of bone^33,34^.

Diabetes affects the growth of microcracks as longer microcracks were found in this study at 24 weeks of diabetes when compared with its control group p < 0.05 and with 4 and 12-week duration of diabetes p < 0.01 and p < 0.05 respectively. However, as we did not quantify the microcracks longer than 100 µm the data presented in this study (Table 1) may underestimate the true crack density and lengths of microcrack. Previous studies have shown that the length of a microcrack is a critical factor in determining the risk of failure, and anisotropic bone microstructure allows the initiation of microcracks but acts as a barrier to its propagation^22^.

Crack numerical density was significantly higher (p < 0.001) at 4 weeks of a duration of diabetes when compared with its respective control and with 12 (p < 0.05) and 24 (p < 0.01) weeks after the onset of DM. However, Crack surface density was highest at 4 (p < 0.01) and 24 weeks (p < 0.05) of a duration of diabetes when compared with their respective controls but no statistically significant difference is seen in groups between different duration of DM.

Investigation of bone microstructure using TEM in this study was helpful in the further interpretation of the results obtained. Collagen fibres are arranged tightly within the trabaculae and the presence of different directions of these fibres within trabeculae is most likely to arrest the growth of microcracks. Separation of collagen fibrils resulted in the formation of meshwork mostly found at 12 weeks of diabetes, and the appearance of multiple sub-micron size voids. These small voids appeared as diffuse damage under fluorescence microscopy. These meshes hence act as a barrier and most likely tend to absorb the forces and arrest the growth of microcracks. Alterations in the normal pattern of collagen fibrils were detected in all diabetic bone specimens. Previous studies^34^ have also shown changes in bone structure, analyzed with both microcomputed tomography (μCT) and histomorphometry, such as lower bone volume and fewer and thinner trabeculae and increased porosity in streptozotocin-induced type 1 diabetic animal models.

Crack numerical density was highest at an early stage of diabetes as numerous small microcracks were formed due to ultrastructural changes in bone such as fibre/matrix debonding or separation and sliding of mineralized collagen fibres^34^. These small linear microcracks may be able to initiate the remodeling cascade and get repaired through the targeted remodeling. Decreased crack numerical density in the later stage of diabetes can be explained due to changes found in the collagen fibril arrangement. Additionally perhaps due to a decreased bone formation as reported in earlier studies^18,35^, some of the microcracks formed at an earlier stage of diabetes are unable to get repaired and they propagate to longer lengths as the disease progresses. The altered bone microstructure as shown in Fig. 5f also favors longer microcracks as observed at 24 weeks of a duration of diabetes. Collagen fibrils were widely separated in a haphazard manner and large areas of afibrillar matrix dominate the bone microarchitecture. Previous studies^36^ also have demonstrated a reduction in collagen synthesis in diabetic rats.

The mineral apposition rate i.e the rate at which mineral accretion occurs at a remodeling site during the period of bone formation was depressed in all diabetic samples compared to the controlled ones which indicate low osteoblast activity and deficient repair mechanisms in diabetes^37^ leading to progression of microcracks at the late stage of diabetes.

Preliminary data from our earlier study showed low levels of serum osteocalcin and bone alkaline phosphatase and increased levels of pentosidine and CTx levels in DM1^38^. It was shown that chronic hyperglycaemic state in type I DM impairs bone remodeling by decreasing bone formation and increasing bone resorption. An increase in advanced glycation end products also contributes to diabetic osteopathy^38^. The alterations to non-collagenous proteins (NCPs), accumulation of advanced glycation end-products and additionally increase apoptosis of osteocytes may have affected the crack initiation and growth in DM1^31,39–41^.

NCPs interact with collagen fibrils and may function as “glue” by preventing the separation of the mineralized collagen fibrils, and thereby counteract the formation of cracks and enhance the bone’s resistance to fracture^32^. Osteocalcin is the most abundant non-collagenous protein of the bone matrix. Bones without osteocalcin are more porous and showed elongated and broken ligament bridges between the mineralized collagen fibrils^41^. Low serum osteocalcin levels are reported at a very early stage of DM1^42,43^ hence this could be one of the factors contributing to the increased number of microcracks seen in diabetes.

AGEs represent several intermolecular crosslinks that are formed as a result of non-enzymatic glycation (NEG). AGEs accumulation occurs in diabetes^39,44^ and has been shown to modify the matrix proteins in bone^39,40,43^.

Additionally, disruption of the structure of the bone cells may have contributed^15^ to the propagation of microcracks as it has been shown by Lai *et al*.^41^ that an increase in the number of apoptotic osteocytes occurs in DM1 which increases the density and length of microcracks^45^.

Bone microstructure demonstrates a toughening mechanism by dissipating the energy in the form of an increased number of diffuse damage areas which were found predominantly in 12 weeks of a duration of diabetes compared to control. They were observed within the trabeculae however unlike other studies^46^ we did find both linear and diffuse damage in the same region of bone as well as in different regions of bone. The formation of diffuse damage areas probably is protective in halting the formation of longer microcracks.

Thus in early diabetes, microstructural features in bone are still acting as a barrier to crack propagation. The formation of areas of diffuse damage enables the bone matrix to confine microdamage to a limited region by dissipating the energy which otherwise would result in the formation of linear microcracks which may propagate to cause failure. The presence of diffuse damage is considered as a superior toughening mechanism over linear microcracks, that averts failure in bone by dissipating energy and delays crack propagation^47^. Diffuse damage also naturally occurs as a result of physiological loading just like linear microcracks^26^. It has also been demonstrated in this study that the ability of bone to form diffuse damage is less in the late stage of diabetes as the microstructure of bone is completely distorted and collagen fibrils are spread wide apart. Others have shown that aging also depresses diffuse damage in bones^33^.

Bone is a hierarchically structured porous composite material consisting of type I collagen, mineral, water, and non-collagenous proteins. It allows bones to withstand complex mechanical loading and resist fractures. While rats are frequently used to study osteoporosis it is important that bone site and age of the animal must be such that remodeling is the predominant activity^48^. Accumulation of microdamage is documented by several studies contributing to the deterioration of bone quality and has a negative effect on bone toughness or resistance to fracture^8,26,46,49^.

## Conclusion

Type 1 DM predisposes to increased fracture risk which cannot be explained alone by the decrease in bone mineral density, changes in bone quality also play a significant role in skeletal fragility associated with diabetes. This study has investigated bone microdamage as an important feature of bone quality in rats with induced DM1. The study shows that DM1 alters bone architecture at an early stage in diabetes, which favors the initiation of microcracks. The heterogeneous bone microstructure favors the toughening mechanisms in early diabetes through diffuse damage and arrests the propagation of linear microcracks by bridging, splitting and deflecting when it comes across a microstructural feature. Moreover, perhaps due to a decrease in bone anabolic function and altered bone remodeling cycle as reported in DM1^34,38,42^ microcracks accumulate and propagate to longer lengths in later stages of DM, compromising bone mechanical properties and eventually fracture. Microdamage should be considered as an important factor in the context of bone quality and fracture risk.

## Methods

### Ethical statement

Thirty adult male Wistar rats, weighing between, 270 and 300 g, were obtained from the Animal House Facility at United Arab Emirates University (UAEU) for this study. All experiments and procedures were carried out according to the National Institute of Health (NIH) guidelines for the care and use of laboratory animals and approved by the appropriate local or national ethics board from the Animal Research Ethics Committee of the College of Medicine and Health Sciences, UAE University.

The animals were singly housed in cages under the standard conditions with a 12 h alternating light and dark cycle, 22–24 °C, in 50–60% humidity and provided with free access to standard rat chow and water *ad libitum* during the two weeks of acclimatization and for the experimental period. All efforts were made to minimize animal suffering and to reduce the number of animals used.

Thirty animals were equally divided into control and experimental groups. Experimental diabetes mellitus was induced in 15 normal adult Wistar rats by a single intraperitoneal injection of streptozotocin [STZ, Santa Cruz (U-9889) 60 mg/kg body weight] dissolved in a freshly prepared citrate buffer (0.1 M, pH 4.5)^50^. Equal volumes of the vehicle were injected into the 15 control rats. In order to study the effect of DM at 4, 12 and 24 weeks after the onset of DM, animals were further sub-divided into six groups control (A, B, and C) and diabetic groups (A-DM 4 week, B-DM 12 week and C-DM 24 week of induced diabetes) n = 5 for each group. Blood was withdrawn from the tail vein from all rats immediately before streptozotocin injection and daily afterwards to check for the blood glucose levels using a blood glucose meter (Accu-Chek Performa; Roche Diagnostics, USA). Animals with fasting blood glucose levels of more than 16.7 mmol/l were as diabetic. Normoglycemia (5–6.7 mmol/l) was confirmed in the control animals. The blood glucose concentrations and body weight were monitored fortnightly. Clinical diabetic signs such as polyphagia, polydipsia, polyuria, and body weight loss were also monitored.

All animals were injected subcutaneously (S/C) with fluorochrome dyes. Alizarin red was injected 30 mg/kg body weight before the induction of diabetes. Calcein green (15 mg/kg body weight) and xylenol orange (90 mg/kg body weight) were injected at two-week intervals^51^ in each group. Rats were euthanized three days after the injection of the last label at 4, 12 and 24 weeks of a duration of diabetes with their matched controls.

#### Preparation of specimens for fluorescence and confocal microscopy

Femurs were dissected out for the study. The neck of the right femur was cut using a diamond saw (Minitom, Struers) and processed for methylmethacrylate embedding and sectioning using a previously described protocol^52^. Briefly, bone specimens were fixed in ethyl alcohol, dehydrated in graded alcohols (70–100%) to facilitate uniform polymerisation of the resin. Following dehydration, specimens were infiltrated and embedded with methylmethacrylate (MMA) solution (MMA, dibutylphthalate and benzoyl peroxide). The bone specimens were placed in glass vials containing the solution in a vacuum desiccator for three days before being heated in an oven at 55 °C for further three days. Blocks were trimmed using a diamond saw (Minitom, Struers), with 12.7 cm × 0.4 mm blade (Bueher, Lake Bluff, IL). Longitudinal sections of 10–30 μm were taken using a Leica RM 2265 microtome and thicker longitudinal sections of 250 µm were also cut using a diamond saw (Minitom, Struers), and hand-ground 100–150 µm. The sections were air-dried on filter paper and then mounted on a glass slide using DPX mounting medium (O.Kindler, E. Germany) and covered with a coverslip.

The cross-sectional area of the sections taken from the trabecular bone was measured and microcracks were identified and analyzed using established criteria^25^. Additionally, the rate of mineral apposition (MAR) was calculated by dividing the distance between the fluorescent markers by the time interval between their administration^53^ and the data is presented in Table 2. Slides were analyzed using an Olympus Research Inverted microscope Model IX53 with fluorescence attachment. Images were captured using a digital camera model DP73 with “cells sens entry” software. Combination filter cube ET-DAPI/FITC/TRITC 69000, and separate filters FITC and TRITC were used to capture images.

The morphology of all the microcracks found was further confirmed by analyzing them with laser scanning confocal microscopy technique using Nikon 80i C1 (Nikon, Kyoto, Japan); laser scanning confocal microscope (LSCM) equipped with a krypton-argon laser. The microscope was operated at an excitation wavelength of 488 nm with a bandpass filter of 480 nm/25 nm and an excitation wavelength of 543 nm, with a bandpass filter of 515/30 nm, respectively. A series of images were taken throughout the depth of a microcrack using an LSCM. The thickness of the slice/step size for Z-stacking was 1–6 µm and the depth through which the bone was imaged was limited to a maximum of 30 µm for thinner and 100 µm for thicker hand-ground longitudinal sections in order to avoid bone tissue attenuation of both the laser beam penetration and the emission light. The length of recorded microcracks was limited to 100 µm as previous studies have shown the mean length of microcracks formed *in vivo* less than 100 µm^53,54^.

Typical linear microcracks, as well as areas of diffuse damage, were analyzed for both control and diabetic specimens. Crack data were expressed in terms of mean crack length (Cr.Le) (µm); crack numerical density; total number of cracks occurring per mm^2^ (Cr.Dn); crack surface density: total length (µm) of crack per mm^2^ (Cr.S.Dn) and diffuse damage density (Dx.Dn) as the number of diffuse damage areas (Dx.Ar) per mm^2^ of bone area.

#### Preparation of specimens for transmission electron microscopy (TEM)

The left femur from each rat was dissected out and the neck of the femur was prepared for transmission electron microscopy. Briefly, the tissue was washed with 0.1 M phosphate buffer at pH 7.2 and then immersed immediately in freshly prepared Karnovsky’s fixative at pH 7.2^55^ for 24 hours at 4 °C in a refrigerator. Bone specimens were post-fixed in 1% osmium tetroxide for 1 hour followed by decalcification in EDTA (ethylenediaminetetraacetic acid) solution. The specimens were dehydrated in a series of graded ethanol from 70% to 95% and 100% and then finally in propylene oxide. Tissue specimens were infiltrated and embedded in Agar100 epoxy resin and polymerized at 65 °C for 24 hours. Blocks were trimmed and semi-thin and ultrathin sections were cut with an ultramicrotome (Leica EM UC 7, Vienna, Austria). Semi-thin sections (130 nm thickness) on glass slides were stained with 1% aqueous toluidine blue on Electro-thermal slide drying bench at 55 °C and ultrathin sections of golden colour (95 nm) on 200 mesh Cu grids were then contrasted with uranyl acetate, followed by lead citrate^56,57^ double stain. The grids were examined at different magnification with “Tecnai G2 Spirit BioTwin” transmission electron microscope (City, Netherlands).

### Statistical analysis

Data were expressed as mean ± standard deviation (SD). A comparison of results between the two groups was made by the independent samples t-test with a probability value of 95% (p < 0.05 and comparison between multiple groups was performed by using two-way analysis of ANOVA with Bonferroni correction in the Graph Pad Prism 5. A p-value of 0.05 or below was considered statistically significant.

### Data sharing statement

The datasets generated during and/or analyzed during the current study are available from the corresponding author on reasonable request.
